# Toll-Like Receptor 2 Is Required for Inflammatory Process Development during *Leishmania infantum* Infection

**DOI:** 10.3389/fmicb.2017.00262

**Published:** 2017-02-23

**Authors:** Laís A. Sacramento, Jéssica L. da Costa, Mikhael H. F. de Lima, Pedro A. Sampaio, Roque P. Almeida, Fernando Q. Cunha, João S. Silva, Vanessa Carregaro

**Affiliations:** ^1^Department of Biochemistry and Immunology, University of São PauloRibeirão Preto, Brazil; ^2^Center for Biology and Health Sciences, Federal University of SergipeAracaju, Brazil; ^3^Department of Pharmacology, Ribeirão Preto Medical School, University of São PauloRibeirão Preto, Brazil

**Keywords:** *Leishmania infantum*, TLR2, dendritic cell maturation, neutrophil activation, visceral leishmaniasis

## Abstract

Visceral leishmaniasis (VL) is a chronic and fatal disease caused by *Leishmania infantum* in Brazil. Leukocyte recruitment to infected tissue is a crucial event for the control of infections such as VL. Among inflammatory cells, neutrophils are recruited to the site of *Leishmania* infection, and these cells may control parasite replication through oxidative or non-oxidative mechanisms. The recruitment, activation and functions of the neutrophils are coordinated by pro-inflammatory cytokines and chemokines during recognition of the parasite by pattern recognition receptors (PRRs). Here, we demonstrated that the Toll-like receptor 2 (TLR2) signaling pathway contributes to the development of the innate immune response during *L. infantum* infection. The protective mechanism is related to the appropriate recruitment of neutrophils to the inflammatory site. Neutrophil migration is coordinated by DCs that produce CXCL1 and provide a prototypal Th1 and Th17 environment when activated via TLR2. Furthermore, infected TLR2^−/−^ mice failed to induce *nitric oxide synthase* (iNOS) expression in neutrophils but not in macrophages. *In vitro*, infected TLR2^−/−^ neutrophils presented deficient iNOS expression, nitric oxide (NO) and TNF-α production, decreased expression of CD11b and reduced *L. infantum* uptake capacity. The non-responsive state of neutrophils is associated with increased amounts of IL-10. Taken together, these data clarify new mechanisms by which TLR2 functions in promoting the development of the adaptive immune response and effector mechanisms of neutrophils during *L. infantum* infection.

## Introduction

Innate immunity coordinates the immune response against infection through pattern recognition receptors (PRRs), promoting anti-microbial defense mechanisms. Among PRRs, Toll-like receptors (TLRs) are the main studied innate sensors and are grouped into a family of 11 transmembrane proteins (Akira et al., 2006) that specifically recognize different pathogens, including different species of *Leishmania* (de Veer et al., 2003; Janssens and Beyaert, 2003; Muraille et al., 2003; Tuon et al., 2008). TLRs are mainly expressed in phagocytes and presenting cells, such as neutrophils, macrophages, and dendritic cells (DCs), which are the major innate cells that respond to *Leishmania* infection (Liu and Uzonna, 2012; Freitas-Silva et al., 2014; Carlsen et al., 2015). Furthermore, the interactions between TLRs present on leucocytes and *Leishmania* spp. parasites determine the host immune response pattern (Tuon et al., 2008). The protective immune response against *Leishmania* spp. infection is associated with CD4^+^ T cells that produce IFN-γ and IL-17 (Alexander and Bryson, 2005; Ghosh et al., 2013), which stimulate phagocytic cells to express inducible nitric oxide synthase (iNOS) and generate nitric oxide (NO; Swihart et al., 1995; Nascimento et al., 2014), the main mechanism by which intracellular parasites are killed (Liew et al., 1997). Interestingly, TLR agonists induce the release of several pro-inflammatory cytokines, including IL-12 and IL-23, coordinating the development of both Th1 and Th17 subsets (Roses et al., 2008).

Because TLRs provide a bridge between innate and adaptive immunity, their agonists are being tested as a vaccine adjuvant to enhance immune responses and control parasitic infections (Raman et al., 2010; Franklin et al., 2011). We demonstrated that TLR9 plays a critical role in neutrophil recruitment during the protective response against *Leishmania infantum* infection and that it is associated with DC activation (Sacramento et al., 2015). However, the remaining DC activation stage was found even in the absence of TLR9, which affected neutrophil recruitment, suggesting that other TLRs may be required for the host immune response against *L. infantum* infection.

The *Leishmania* spp. surface is covered with molecules recognized by TLRs (Tuon et al., 2008). In this sense, TLR2 has a central role in the recognition of lipophosphoglycan (LPG; Becker et al., 2003; de Veer et al., 2003; Kavoosi et al., 2009), the most abundant molecule present in promastigote forms of different species of *Leishmania* spp., including *L. infantum* (McConville et al., 1992, 1995; Assis et al., 2012), and promotes the host defense against the parasite. As examples, TLR2 recognizes LPG from *L. major* and promotes NK cell activation through NFκB activation (Becker et al., 2003), culminating in the release of reactive oxygen species (ROS) and NO produced by macrophages (Kavoosi et al., 2009, 2010). Accordingly, the injection of a TLR2 agonist as immunotherapy during experimental *L. major* infection prevents lesion development and reduces the parasitic load through a mechanism dependent on activation of DCs and macrophages and production of IL-12 (Huang et al., 2015). Exhausted CD8^+^ T cells from patients with diffuse cutaneous leishmaniasis have their effector mechanisms restored after *in vitro* stimulation with TLR2 ligand (Hernández-Ruiz et al., 2010). *In vivo*, the treatment with TLR2 agonist controls parasite replication in target organs through a mechanism dependent on improvement of CD8^+^ T cells (Bandyopadhyay et al., 2015) and the Th1 response (Chowdhury et al., 2015a) and suppression of CD4^+^ Foxp3^+^ T cell function (Chowdhury et al., 2015b), suggesting that the role of TLR2 during infection is an interesting alternative for restraining parasites from spreading to tissues.

Regarding *L. infantum* infection, there is an association between TLR2 and the production of TNF-α, IFN-γ, and NO after treatment of *L. infantum*-infected patients (Gatto et al., 2015). However, in the aforementioned study, the role of the TLR2 pathway in managing the protective immune response during *in vivo L. infantum* infection was still unclear. To fill this knowledge gap, the goal of this study was to evaluate the function of TLR2 during *L. infantum* infection, focusing on the development of the protective immune response. This work demonstrates that TLR2 displays a protective role in the control of parasite replication in target organs of infection. During infection, TLR2 signaling promotes both activation of DCs and development of the Th1 and Th17 immune response, which together induces neutrophil recruitment to the spleen and liver. In the last instance, neutrophils are activated and produce TNF-α and NO in a TLR2-dependent manner, promoting the restriction of *L. infantum* parasites.

## Materials and methods

### Mice

Female C57BL/6 (wild type; WT) and C57BL/6-TLR2^−/−^ (TLR2^−/−^) mice weighing 18–22 g were housed in the animal facility of the Department of Biochemistry and Immunology, School of Medicine of Ribeirão Preto, University of São Paulo (Brazil), in temperature-controlled rooms (22–25°C) and received water and food *ad libitum*. All experiments were conducted in accordance with the National Institutes of Health (NIH) guidelines for the welfare of experimental animals and with the approval of the Ethics Committee of the School of Medicine of Ribeirão Preto.

### Parasite culture, infection, and load estimation

*L. infantum* (isolate HU-UFS14) was cultured in Schneider medium with 20% heat-inactivated fetal bovine serum, 5% penicillin and streptomycin (both from Sigma-Aldrich, Saint Louis, MO, USA), and 2% male human urine. Parasite virulence was maintained by serial passages in BALB/c mice. Mice were injected in their retro-orbital plexus with 10^7^ stationary-phase *L. infantum* promastigotes in 100 μL of PBS. Hepatic and splenic parasite burdens were determined using a quantitative limiting dilution assay.

### DC generation and infection

Generation of bone marrow-derived cells (BMDCs) was performed as previously described (Carregaro et al., 2008). For the DC activation assay, BMDCs (1 × 10^6^ cells/mL) cultured in RPMI-1640 supplemented with 10% FBS were infected with *L. infantum* promastigotes at a 1:5 ratio (cells/parasites) for 24 h. The supernatants were collected to measure IL-12p40 (BD Biosciences, catalog number 555165) and IL-10 (R&D Systems, catalog number DY417-05) production by an ELISA kit, according to the manufacturer's instructions. BMDCs were harvested from some cultures, and their surface expression was characterized by flow cytometry using antibodies against CD11c, TLR2, MHCII, CD40, and CD86 conjugated to PECy7, PE, FITC, PerCP, APC, respectively, as well as control isotypes.

### Cell culture and inflammatory cell phenotypes

Single-cell suspensions of spleen tissue samples from TLR2^−/−^ or WT mice at the 4th wpi were aseptically prepared, diluted to a concentration of 2 × 10^6^ cells/mL, and dispensed into 48-well plates in a total volume of 500 μl of complete RPMI-1640 medium (1 × 10^6^ cells/well; Gibco) with or without *L. infantum* crude antigen (50 μg/mL). The cell culture supernatants were harvested after 72 h of culture at 37°C in 5% CO_2_ and the IL-17, IFN-γ, and IL-10 levels in the supernatants were determined using commercial ELISA kits. For detection of IFN-γ and IL-17 in the liver, tissue samples were harvested by tissue trimmer, weighed, and titered in 1 mL of PBS Complete (Roche Diagnostics, Mannheim, Germany) containing protease inhibitor cocktail. The levels of cytokines were determined using commercial ELISA kits from R&D Systems for IL-10 and IL-17(catalog number DY417 and DY421, respectively) and BD Biosciences for IFN-γ (catalog number DY485).

For leukocyte identification, spleeny cells isolated from WT and TLR2^−/−^ were stained with fluorescent labeled monoclonal antibody that recognizes a target feature on or in the cell. The inflammatory cells were gated based on their characteristic size (FSC) and granularity (SSC), and the T lymphocytes (CD4^+^CD3^+^), dendritic cell activation markers (CD11c^high^CD40^+^, CD11c^high^CD86^+^, and CD11c^high^MHC-II^+^) and neutrophils (Ly6G^high^MHCII^−^) were identified individually. For intracellular staining, the cells were cultured with PMA (50 ng/mL) and ionomycin for 4 h in order to obtain the maximum cytokine production, permeabilized with a Cytofix/Cytoperm Kit (BD Biosciences) according to the manufacturer's guidelines and stained with anti-IFN-γ or anti-IL-17 conjugated to APC-Cy7 and Alexa Fluor 700 and with anti-CD3 and anti-CD4 for surface staining with FITC and PerCP, respectively. Rat IgG2b and IgG2a were used as the isotype controls. All the antibodies were supplied from BD Biosciences and eBiosciences (San Diego, CA, USA). Cell acquisition was performed using a FACSort flow cytometer. The data were plotted and analyzed using FlowJo software (Tree Star, Ashland, OR, USA). The total leucocyte counts were determined by measuring the relative expression of the leucocyte subpopulations stained with specific antibody in 300,000 acquired events proportional to the leukocyte number obtained in a Neubauer chamber.

### Histopathological and immunohistochemical analyses

The mice were euthanized 0 and 4 weeks after infection, and their livers were removed. The tissues were fixed in formalin, dehydrated in graded ethanol, and embedded in paraffin. Serial sections (5 μm) were cut and mounted on glass slides precoated with 0.1% poly-l-lysine (Sigma-Aldrich). Histological assessment was performed after routine hematoxylin-eosin staining. The area of the liver lesion was determined using Leica Qwin software (Mannheim, Germany).

For immunohistochemical reactions, the paraffin was removed from the tissues, and antigenic recovery was performed by heating the sections in citrate buffer (pH 6.0) for 30 min at 37°C. Endogenous peroxidase was blocked using 3% H_2_O_2_, cells were permeabilized with Triton 0.5%, and non-specific reactions were blocked with 1% bovine serum albumin (BSA). The sections were incubated overnight with a monoclonal rat anti-7/4 antibody (Abcam Plc, Cambridge, UK) or control isotype antibodies (Abcam, Cambridge, MA, USA), followed by incubation with a biotinylated secondary antibody and avidin-biotin complex (Vector Laboratories, Ontario, Canada). The reaction was detected with diaminobenzidine, and the sections were counterstained with Mayer's hematoxylin. For intracellular staining for iNOS (Santa Cruz Biotechnologies, Dallas, TX, USA), liver sections were permeabilized with 0.01% saponin. Afterward, the sections were incubated with an avidin-biotin-peroxidase complex (Vector Laboratories, Ontario, Canada), and the color was developed using 3,3′-diaminobenzidine (Vector Laboratories). The slides were counterstained with Mayer's hematoxylin.

### Quantitative RT-PCR

Total RNA was isolated from neutrophil cultures or from the spleens and livers of WT and TLR2^−/−^ mice at the 4th wpi and uninfected using an SV Total RNA Isolation System Kit (Promega, Madison, WI, USA). Gene expression was normalized to hypoxanthine-guanine phosphoribosyltransferase (HPRT) expression for spleen and glyceraldehyde-3-phosphate dehydrogenase (GAPDH) expression for liver and neutrophil culture. The primer sequences used were as follows: HPRT forward primer, 5′-TGGAAAAGCCAAATACAAAGC-3′, and reverse primer, 5′-CAACATCAACAGGACTCCTCG-3′; GAPDH forward primer, 5′-TGCAGTGGCAAAGTGGAGAT-3′, and reverse primer, 5′-CGTGAGTGGAGTCATACTGGAA-3′; TLR2 forward primer, 5′-AAGTCTCCGGAATTATCAGTCC-3′, and reverse primer, 5′-TGATGGATGTCGCGGAT-3′; and iNOS forward primer, 5′-CGAAACGCTTCACTTCCAA-3′, and reverse primer, 3′-TGAGCCTATATTGCTGTGGCT-5′.

### Neutrophil isolation and culture

For bone marrow neutrophil purification, femurs and tibias were removed and flushed. The bone marrow cells were suspended in Hanks' balanced salt solution (Sigma-Aldrich, St. Louis, MO, USA) and laid on top of a two-layer Percol (Sigma-Aldrich, St. Louis, MO, USA) gradient (72 and 65% in Hanks' balanced salt solution). Mature neutrophils were recovered at the interface of the gradient fraction. For NO, TNF, and IL-10 quantification, neutrophils (5 × 10^5^) were cultured in RPMI-1640 supplemented with 10% FBS in the presence of *L. infantum* promastigote forms at a 1:5 ratio (cells:parasites) for 24 h at 37°C in an atmosphere of 5% CO_2_. The supernatants were harvested to measure NO by the Griess method (Martins et al., 2001) and TNF and IL-10 by an ELISA kit according to the manufacturer's instructions (BD Biosciences, catalog number DY410-05 and DY417, respectively). For neutrophil activation, 1 × 10^6^ neutrophils/well were distributed in 48-well plates in 300 μL of complete RPMI medium in the presence of medium only or *L. infantum* promastigote forms at a 1:5 ratio for 4 h. For the *L. infantum* uptake assay, parasites were previously stained with 1.25 mM carboxyfluorescein succinimidyl ester (CFSE; Sigma-Aldrich, St. Louis, MO, USA) in PBS. The neutrophils were primed with recombinant murine IFN-γ (100 ng/mL) for 1 h and cultured with CFSE-stained *L. infantum* promastigotes for 4 h. Then, the cells were fixed and stained with anti-α-Ly6G and anti-α-CD11b antibodies that were conjugated with APC and PEcy7, respectively (BD Bioscience and eBioscience, San Diego, CA, USA). Cell acquisition was performed using a FACSort flow cytometer. The data were plotted and analyzed using FlowJo software (Tree Star, Ashland, OR, USA).

### Statistical analysis

Data are expressed as the mean ± SEM and are representative of 2–4 independent experiments. The results from individual experiments were not combined because they were analyzed individually. The means from the different groups were compared by ANOVA followed by Tukey's honest significant difference (HSD) test. Comparisons between two groups were determined using Student's *t*-test. Analyses were performed using Prism 5.0 software (GraphPad). Statistical significance was set at *P* < 0.05.

## Results

### TLR2 is induced during *L. infantum* infection and participates in the restriction of parasites into target organs

**Figure 1:**
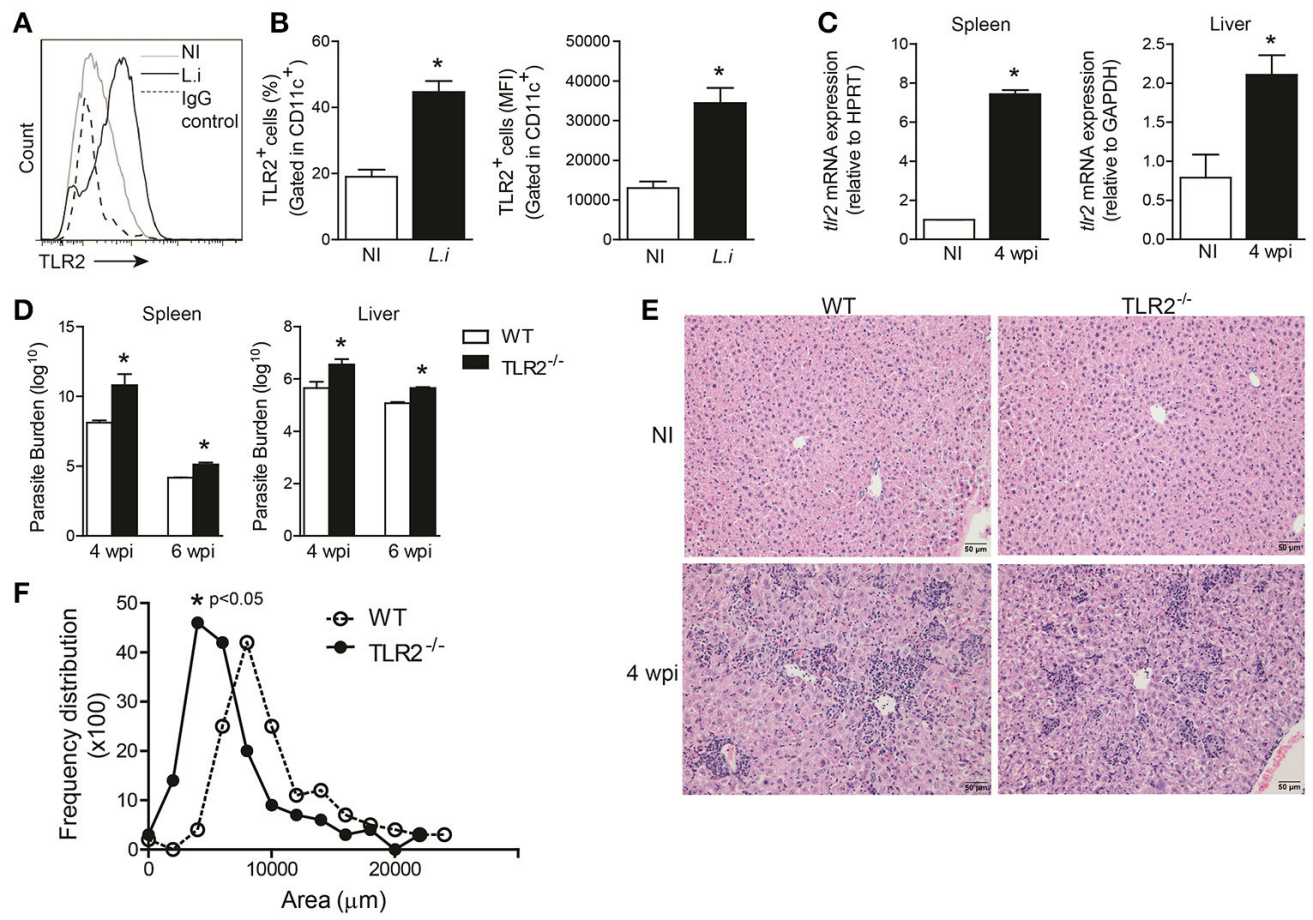
**TLR2 is important for host protection against ***L. infantum*****. TLR2 expression in *in vitro L. infantum*-infected WT BMDCs (1:5) during 24 h **(A)**. The percentage and MFI of DC (CD11c^high^) expression of TLR2 was determined by flow cytometry **(B)**. *tlr2* mRNA expression in the spleens and livers from WT mice at the 4th wpi was evaluated by real-time PCR and normalized to the constitutively expressed HPRT and GAPDH genes, respectively **(C)**. Parasite burdens in the spleen and liver were determined in WT and *TLR2*^−/−^ mice at the 4th and 6th weeks p.i. by limiting dilution **(D)**. Representative photomicrography of stained liver sections is shown at 20 times the original magnification **(E)**. The inflammation areas in WT (open circles) and TLR2^−/−^ (closed circles) mice were quantified using Leica Qwin software and are shown as the frequency distribution of the lesion areas **(F)**. Data are expressed as the mean ± SEM and are representative of three independent experiments. ^*^P < 0.05 [Student's *t*-test **(B,C,F)** or two-way ANOVA with Tukey's *post-hoc* test **(D)**] is relative to the control group.

It has been suggested that TLR2 interacts with *Leishmania* species, including *L. infantum*, and triggers the host immune response against the parasite (de Veer et al., 2003; Assis et al., 2012). First, we investigated whether TLR2 expression was altered in BMDCs infected with promastigote forms of *L. infantum*. During infection, both the percentage and the mean fluorescence intensity (MFI; Figure 1B) of TLR2 expression on CD11c^+^ cells were increased. Furthermore, *tlr2* mRNA expression in the spleen and liver from infected WT mice at the 4th week was significantly increased, *p* = 0.002 and *p* = 0.001, respectively (Figure 1C), demonstrating that *L. infantum* infection modulates TLR2 expression both *in vitro* and *in vivo*.

To assess the role of TLR2 in the control of parasite growth, TLR2^−/−^ mice and control littermates were infected with 10^7^ promastigote forms of *L. infantum*, and parasite loads in their spleens and livers were quantified after different time-points of infection. TLR2^−/−^ mice harbored more parasites in both target organs during weeks 4 and 6 after infection than WT animals (Figure 1D).

The control of infection during experimental VL is related to leukocyte infiltration of the liver, generating granulomas (Stanley and Engwerda, 2006). Thus, in order to determine the factors involved in the susceptibility of infected TLR2^−/−^ mice, the amount of inflammatory cell infiltration in the liver of WT and TLR2^−/−^ mice at the 6th wpi and naïve mice, as a control, was determined. Histologic examination of livers demonstrated that WT mice exhibited large areas of inflammatory tissue at the 4th wpi. In the absence of TLR2, the inflammatory infiltrate was dispersed in the hepatic parenchyma, which presented reduced areas of leucocyte infiltration (Figure 1E). Confirming the histopathology analyses, the quantification of inflamed areas in hepatic tissue demonstrates smaller areas of inflammatory infiltrate in the TLR2^−/−^ mice. While the average size of inflammatory areas in the WT group was 8,000 μm^2^, the average size of the inflammatory areas in TLR2^−/−^ mice was 5,000 μm^2^, *p* < 0.05 (Figure 1F), suggesting that TLR2 contributes to inflammatory infiltration during VL.

### TLR2 induces the development of Th1 and Th17 protective immune responses and promotes DC activation during *L. infantum* infection

**Figure 2:**
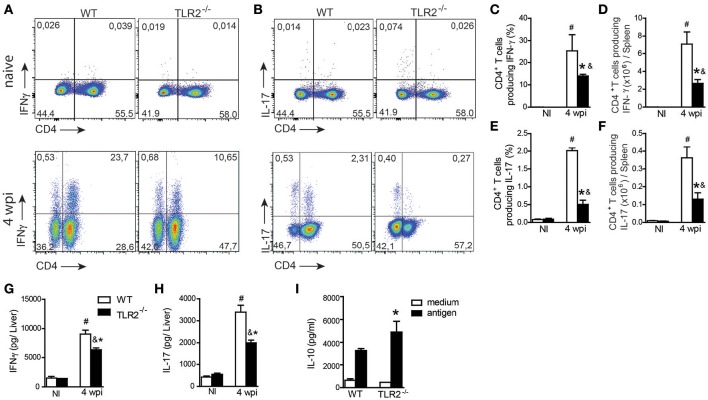
**TLR2 participates in Th1 and Th17 immune response development during ***L. infantum*** infection**. The spleen cells from uninfected (NI) or 4 wpi WT and TLR2^−/−^ mice were restimulated *in vitro* with PMA (50 ng/mL) and ionomycin (500 ng/mL) for 4 h and analyzed for intracellular cytokine production by flow cytometry. Dot plots represent the frequency of naïve (above) and infected (below) CD4^+^ T cell-producing IFN-γ **(A)** and IL-17 **(B)**, and the graph bars represent the percentage **(C,E)** and the absolute number **(D,F)** of these cells. The liver fragments from the WT or TLR2^−/−^ mice at the 4th wpi were collected and weighed for the determination of IFN-γ **(G)** and IL-17 **(H)** by ELISA in the homogenate supernatants. *In vitro* restimulation of spleen cells with the *L. infantum* antigen (50 μg/mL) or medium for 72 h, and the levels of IL-10 **(I)** were measured in the culture supernatants by an ELISA. Data are expressed as the mean ± SEM and are representative of three independent experiments, *N* = 4–5. ^*^P < 0.05 compared to infected WT **(A–G)** or compared to antigen-stimulated WT **(H)**, *#P* < 0.05 compared to uninfected WT, &*P* < 0.05 compared to uninfected TLR2^−/−^ (two-way ANOVA with Tukey's *post-hoc* test).

We have previously shown that TLR9 pathway signaling, in addition to modulating innate immunity, drives the adaptive immune response controlling parasitic spread (Sacramento et al., 2015). To determine whether this activation mechanism induced by TLR9 is shared with other TLR ligands, we determined both Th1 and Th17 responses in target organs of infection. Spleen cells from naïve and infected WT and TLR2^−/−^ mice were restimulated *in vitro* with polyclonal PMA plus ionomycin, and intracellular cytokine production was analyzed. *L. infantum* infection promoted both Th1 and Th17 patterns of immune response in WT and TLR2^−/−^ mice compared with respective littermate controls. However, significant reductions in the frequencies and absolute numbers of both IFN-γ (Figures 2A,C,D)- and IL-17 (Figures 2B,E,F)-producing CD4-T cells were observed in infected TLR2^−/−^ mice compared with those in infected WT mice. In addition, liver samples from infected TLR2^−/−^ mice contained significantly lower levels of IFN-γ (Figure 2G) and IL-17 (Figure 2H) compared with those of WT mice. IL-10 production was measured in cultured spleen cells in response to stimulation with parasite antigen and, consistent with a reduction in pro-inflammatory cytokines, was significantly increased in the supernatant of cultured cells lacking TLR2 compared with the littermate control (Figure 2I), suggesting that TLR2 signaling modulates cytokines during VL.

**Figure 3:**
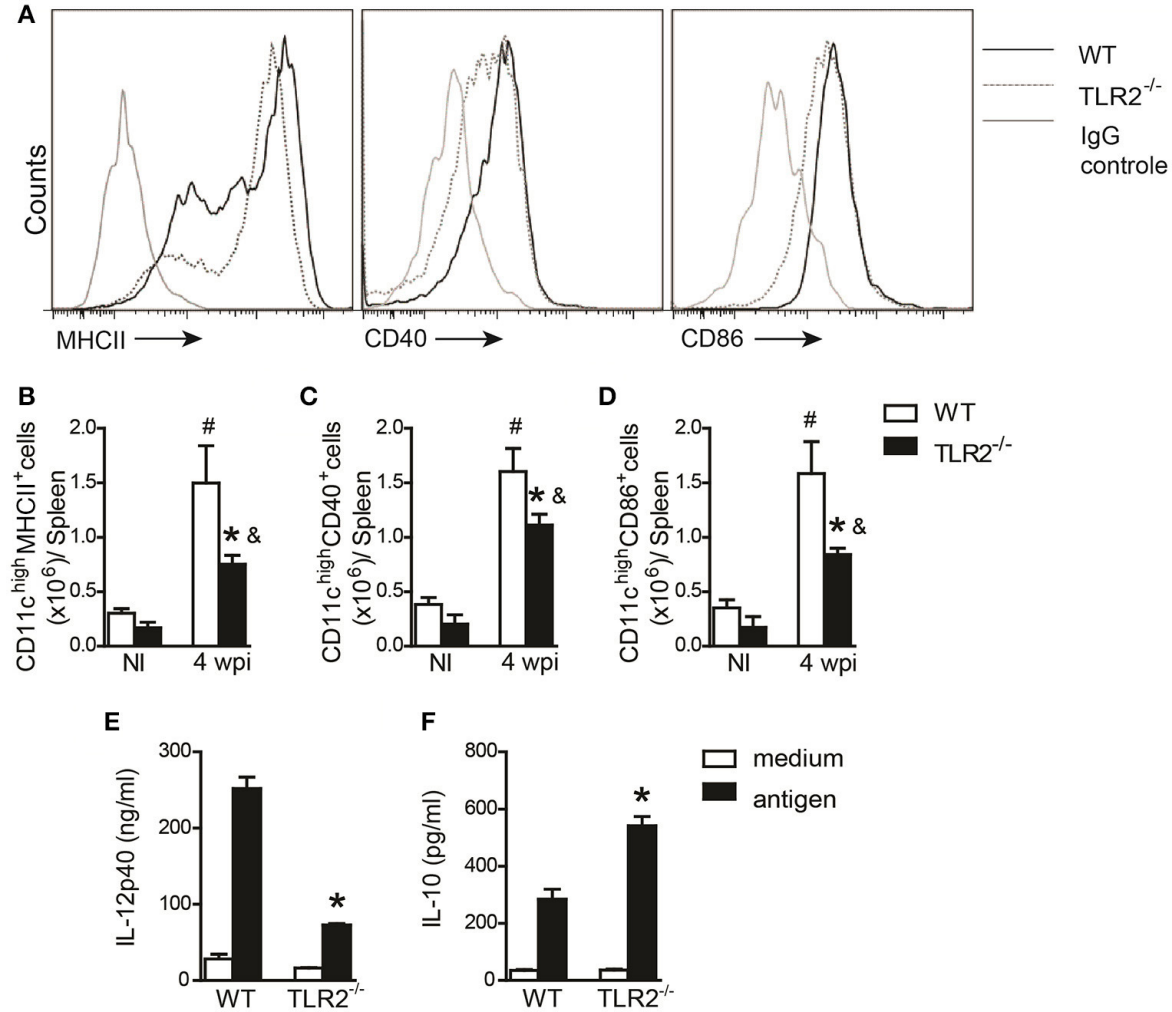
**DC activation during ***L. infantum*** infection is dependent on TLR2**. *In vivo* surface markers of DCs from uninfected (NI) and at 4th wpi WT and TLR2^−/−^ mice were determined by flow cytometry. Histograms demonstrate the costimulatory molecules in the CD11c^high^ population **(A)** and bar graphs represent the percentage of MHCII **(B)**, CD40 **(C)**, and CD86 **(D)**. All analyses were performed on CD11b^+^CD11c^high^ gated cells. WT and TLR2^−/−^ BMDCs were infected with *L. infantum* (5:1) or medium for 24 h and IL-12p40 **(E)** and IL-10 **(F)** levels in the supernatants were measured by ELISA. Data are expressed as the mean ± SEM and are representative of three independent experiments, *N* = 4–5. ^*^P < 0.05 compared to infected WT **(B–D)** or compared to stimulated WT **(E,F)**, *#P* < 0.05 compared to uninfected WT, &*P* < 0.05 compared to uninfected TLR2^−/−^ (two-way ANOVA with Tukey's *post-hoc* test).

DC maturation and inflammatory cytokine secretion are important for modulating CD4^+^ T cell polarization (von Stebut et al., 2000). Therefore, we evaluated the activation profile of DCs from the spleens of WT and TLR2^−/−^ mice at the 6th wpi or of naïve mice. Our data demonstrated impairment of MHCII (Figures 3A,B), CD40 (Figures 3A,C), and CD86 (Figures 3A,D) expression on CD11c^+^ cells recovered from infected TLR2^−/−^ mice compared to infected WT mice. As a consequence of the failure of DC maturation, *in vitro*-infected BMDCs derived from TLR2^−/−^ mice infected with *L. infantum* presented reduced amounts of IL-12p40 (Figure 3E) and elevated secretion of IL-10 (Figure 3F) into the supernatant compared to WT BMDCs. Taken together, these results suggest that the absence of TLR2 committed the DC activation profile, affecting both Th1 and Th17 immune response development possibly due to a detrimental effect on IL-10 production.

### TLR2 promotes neutrophil recruitment to target organs of VL

**Figure 4:**
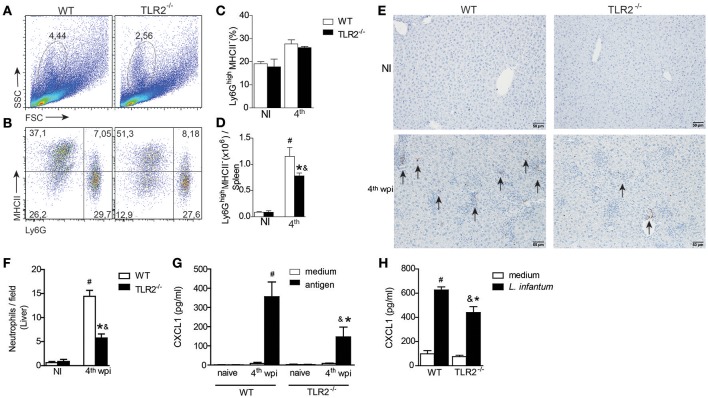
**TLR2 induces neutrophil migration to target organs of ***L. infantum*** infection**. The neutrophil population was gated based on their characteristic size (FSC) and granularity (SSC) **(A)**. The dot plots represent the frequency of neutrophil population characterized by Ly6G^+^MHCII^−^ by flow cytometry **(B)**. The bar graphs represent the percentage **(C)** and absolute number **(D)** of the Ly6G^+^MHCII^−^ population in the spleens from WT and TLR2^−/−^ mice at the 4th wpi or from uninfected mice (NI). Neutrophil numbers in liver sections of WT and TLR2^−/−^ mice at the 4th wpi or from uninfected mice (NI) by immunohistochemistry with anti-mouse 7/4 antibody arrows indicate 7/4^+^ cells **(E)**. Quantification of 7/4^+^ cells present in the liver tissue of WT and TLR2^−/−^ mice at the 4th wpi or from NI mice **(F)**. Arrays show the 7/4^+^ cells. CXCL1 measured from the supernatant of *in vitro-*restimulated spleen cells with the *L. infantum* antigen (50 μg/mL) or medium for 72 h **(G)** or from *L. infantum in vitro*-infected DCs **(H)** by ELISA. Data are expressed as the mean ± SEM and are representative of three **(A–D)** and two **(E,F)** independent experiments, *N* = 4–5. ^*^P < 0.05 compared to infected WT **(D,F)** or compared to stimulated WT **(G)**, *#P* < 0.05 compared to uninfected WT, &*P* < 0.05 compared to uninfected TLR2^−/−^ (two-way ANOVA with Tukey's *post-hoc* test).

Neutrophils are recruited to *Leishmania* inoculation foci (Thalhofer et al., 2011) and participate in the restriction of parasites in target organs of VL (Smelt et al., 2000; Rousseau et al., 2001; McFarlane et al., 2008; Sacramento et al., 2015). To evaluate the contribution of TLR2 in the recruitment of neutrophils during *L. infantum* infection, we characterized the neutrophils present in spleens and livers from infected WT or TLR2^−/−^ mice. Based on the characteristic size (FSC) and granularity (SSC), we observed a significant reduction in the percentage of granulocytes gated from infected TLR2^−/−^ mice compared to infected WT mice (Figure 4A). We also observed relatively minor differences in terms of the percentage of Ly6G^+^MHCII^−^ cells (Figures 4B,C), most notably, a significant reduction in the total number of this population in infected TLR2^−/−^ mice relative to the littermate controls (Figure 4D).

Regarding neutrophils in the liver, immunohistochemical analysis demonstrated less 7/4-stained cells in the liver sections from infected TLR2^−/−^ mice compared to infected WT mice (Figure 4E). We quantified the staining and identified a drastic drop, ~60%, in the number of neutrophils in the liver sections from infected TLR2^−/−^ mice compared to those from infected WT mice (Figure 4F). The reduction in the number of neutrophils in the absence of TLR2 was concomitant with reduced production of CXCL1, a neutrophil chemotactic mediator, by splenic cells restimulated with parasite antigen (Figure 4G). Consistent with the failure of neutrophil migration and the reduced DC activation profile (Figures 3B–D), we found that bone marrow-derived dendritic cells (BMDCs) from TLR2^−/−^ mice presented a deficiency in the secretion of CXCL1 when infected with *L. infantum* (Figure 4H). All together, these data indicate the participation of TLR2 signaling in DC-mediated neutrophil recruitment to inflammatory foci of VL.

### TLR2 influences iNOS expression in target organs of VL

**Figure 5:**
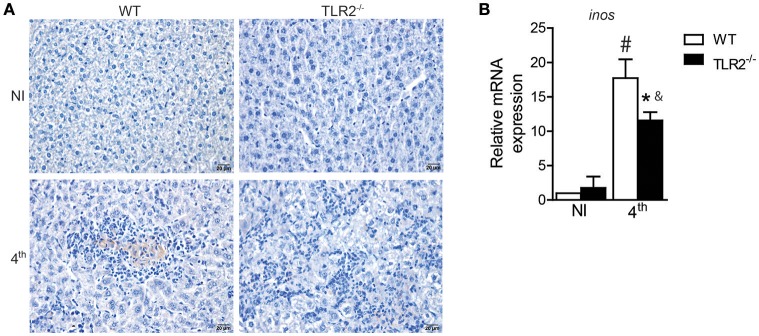
**iNOS expression in target organs of ***L. infantum*** infection is dependent on TLR2**. iNOS staining in liver sections of WT and TLR2^−/−^ mice at the 4th wpi or from uninfected mice (NI) by immunohistochemistry with anti-mouse iNOS antibody **(A)**. *inos* mRNA expression in the spleen from WT mice at the 4th wpi was evaluated by real-time PCR and normalized to the constitutively expressed HPRT **(B)**. Data are expressed as the mean ± SEM and are representative of three **(A)** and two **(B)** independent experiments, *N* = 4–5. ^*^P < 0.05 compared to infected WT, *#P* < 0.05 compared to uninfected WT, &*P* < 0.05 compared to uninfected TLR2^−/−^ (two-way ANOVA with Tukey's *post-hoc* test).

Interleukin 17A acts synergistically with Interferon-γ to promote protection against *L. infantum* infection through iNOS expression and NO production (Nascimento et al., 2014). Because the absence of TLR2 affected both Th1 and Th17 development, we therefore investigated iNOS expression in target organs of VL from infected TLR2^−/−^ mice and littermate controls. The immunohistochemical qualitative analysis demonstrated that *L. infantum* infection induces iNOS expression in the livers of both WT and TLR2^−/−^ mice; however, decreased staining areas for iNOS were observed in the liver sections of infected TLR2^−/−^ mice relative to infected WT mice (Figure 5A). Similar effects were also observed in the spleens of mice that lacked TLR2 that presented reduced expression of mRNA for *inos* compared to infected WT mice (Figure 5B), indicating the involvement of TLR2 in the induction of iNOS expression during VL.

### TLR2 mediates NO production, neutrophil activation, and *L. infantum* uptake

**Figure 6:**
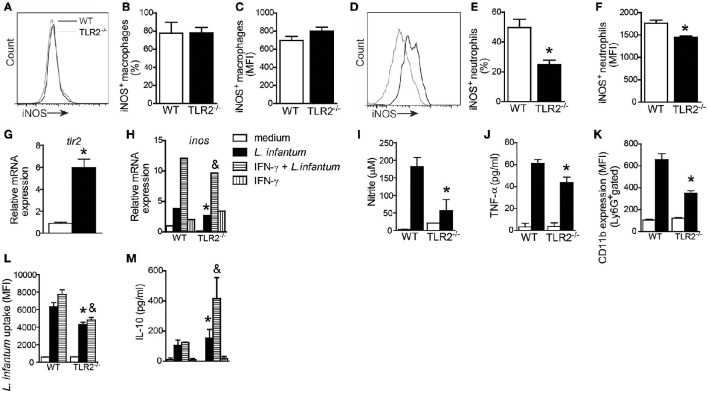
**TLR2 promotes nitric oxide and TNF production by neutrophils and killing of ***L. infantum*****. Spleen cells of WT and TLR2^−/−^ mice were isolated from infected mice and stained for iNOS in CD11c^−^MHCII^+^CD11b^+^ cell (macrophage) and Ly6G^+^MHCII^−^ cell (neutrophil) populations. Representative histograms demonstrate the iNOS expression gated in CD11c^−^MHCII^+^CD11b^+^ cell **(A)** and Ly6G^+^MHCII^−^ cell **(D)** populations. The bar graphs represent the percentage and MFI of iNOS^+^ macrophages **(B,C)** and iNOS^+^ neutrophils **(E,F)**. Neutrophils were isolated from bone marrow and infected with promastigote forms of *L. infantum* (5:1) or uninfected (medium). After 4 h, *tlr2* **(G)** and *inos* **(H)** mRNA expression was analyzed by quantitative PCR, and the supernatant was collected for nitric oxide **(I)** and TNF **(J)** production, or the cells were harvested for CD11b to evaluate the neutrophil activation by flow cytometry **(K)**. For the *L. infantum* uptake assay, the neutrophils were previously primed with IFN-γ (100 ng/mL) for 1 h and cultured with CFSE stained *L. infantum* promastigote forms (5:1) for 4 h **(L)** and IL-10 from the supernatant was measured **(M)**. Data are expressed as the mean ± SEM. ^*^P < 0.05 compared to infected WT **(E,F)** or WT stimulated with *L. infantum* **(G–M)**, &*P* < 0.05 compared to WT stimulated with IFN-γ + *L. infantum* **(H,L,M)** [Student's *t*-test **(E–G)** or two-way ANOVA with Tukey's *post-hoc* test **(H–M)**] relative to the control group.

During *Leishmania* infection, different cell types such as macrophages, inflammatory monocytes, and neutrophils produce NO-dependent iNOS expression, participating actively in the control of parasites (Liew et al., 1991; Wei et al., 1995; Olekhnovitch and Bousso, 2015). To determine which cellular subtype is the primary source of NO in a TLR2-dependent manner, we evaluated iNOS expression in splenic macrophages (CD11b^+^CD11c^−^Ly6G^−^ cells) and neutrophils (CD11b^+^Ly6G^+^MHCII^−^ cells) from infected WT and TLR2^−/−^ mice (Figures 6A,D). Interestingly, the absence of TLR2 on macrophages affected neither the percentage (Figure 6B) nor MFI (Figure 6C) of iNOS expression in macrophages. However, we found a reduction in iNOS expression in neutrophils from mice that lacked TLR2 compared with WT neutrophils. The reduction in iNOS expression was ~50% (Figure 6E) in terms of percentage and 20% in terms of MFI (Figure 6F).

Having determined that TLR2 plays an important role in iNOS expression by neutrophils, we evaluated whether, similar to DCs (Figure 1A), *L. infantum* infection may induce TLR2 expression on neutrophils. Thus, isolated bone marrow-derived neutrophils from WT mice were cultured with promastigote forms of *L. infantum* for 4 h, and the cells were harvested for *tlr2* mRNA expression by quantitative PCR. We observed that the infection induces an ~7.4-fold increase in *tlr2* mRNA expression (Figure 6G). Confirming the importance of TLR2 in iNOS expression, we observed that *in vitro* neutrophils from TLR2^−/−^ mice infected with *L. infantum* presented a reduction in mRNA expression for *inos* compared to WT neutrophils. Even in the presence of IFN-γ, the expression of *inos* mRNA was not induced (Figure 6H). Consistently, the amounts of NO measured in supernatant cultures from TLR2^−/−^ neutrophils were significantly reduced by ~70% compared with WT neutrophils (Figure 6I). In addition, infected TLR2^−/−^ neutrophils presented a significant reduction in the amount of TNF-α compared to WT neutrophils (Figure 6J). Because stimulation of TLRs modulates neutrophil activation by up-regulating integrins (Sabroe et al., 2003), we evaluated the expression of CD11b in infected WT and TLR2^−/−^ neutrophils. Flow cytometric analysis of Ly6G^+^MHCII^−^ cells demonstrated an ~50% reduction in the MFI of CD11b expression in TLR2^−/−^ neutrophils relative to WT neutrophils (Figure 6K).

To extend these findings, the phagocytosis capacity of TLR2^−/−^ neutrophils was evaluated. For this purpose, WT and TLR2^−/−^ bone marrow neutrophils were previously primed with IFN-γ and then infected with CFSE-stained *L. infantum* promastigote forms for 4 h. The TLR2^−/−^ neutrophils presented a reduction in parasite uptake compared to WT neutrophils (*P* < 0.01), even when primed with IFN-γ (*P* < 0.003; Figure 6L). In agreement with these findings, TLR2^−/−^ neutrophils presented increased amounts of IL-10 (Figure 6M). These data demonstrate the participation of TLR2 in neutrophil activation, TNF-α production and NO release, consequently interfering with the parasitic uptake ability.

## Discussion

This study reveals the important role played by TLR2 in the control of *L. infantum* infection. TLR2 is induced during infection and is important for DC activation, which influences the drive of naïve CD4^+^ T cells to the Th1 and Th17 pattern as well as the production of CXCL1. In the last instance, neutrophils are recruited to target organs of infection to restrict parasite replication. Furthermore, TLR2 present on neutrophils has a relevant function in promoting their activation, iNOS expression, NO production and TNF release, restricting the spread of parasites.

It has been shown that TLRs have an essential function during parasitic infections, and several preclinical and clinical studies have explored the use of TLR agonists as adjuvants to reinforce the adaptive immune response during vaccination (Maisonneuve et al., 2014; Ashour, 2015). From an immunotherapeutic perspective, the engagement of TLR2 during *L. infantum* infection appears to be a targeted approach. Using mice genetically deficient in TLR2, we demonstrated that TLR2 is involved in key components of the resistance profile against *L. infantum* infection, such as activation of DCs and production of IL-12, development of the Th1 and Th17 immune response, and recruitment and activation of neutrophils, leading to iNOS expression and NO production and parasitic death.

Although, the contribution of Th1 and Th17 patterns have been well-established during *Leishmania* infection (Engwerda and Kaye, 2000; Ghosh et al., 2013), the link between *Leishmania* parasites and the development of the host protective immune response remains unclear. DCs are the main sensors of microbial patterns and play a central role in the interface between the innate and adaptive immune response through the TLR signaling pathway. In this sense, our results demonstrate the importance of TLR2 for DC activation and, subsequently, for governing the outcome of the host immune response (i.e., modulating the development of both Th1 and Th17). In support of our results, the *in vivo* neutralization of TLR2 during *L. major* infection has been shown to reduce the expression of costimulatory molecules on DCs (Komai-Koma et al., 2014). In contrast, DCs from TLR2^−/−^ mice infected with *L. braziliensis* were more competent in priming naïve CD4^+^ T cells *in vitro*, promoting *in vivo* increases in IFN-γ production and resistance to infection. All together, these studies demonstrate that different *Leishmania* species can modulate the DC activation profile and T cell immune response through TLRs.

Previously, our group demonstrated that TLR9 participates in the restriction of *L. infantum* parasites, contributing to a resistant profile (Sacramento et al., 2015). Based on the present study, we suggest that TLR2 and TLR9 may act cooperatively to induce host protection during *in vivo L. infantum* infection. We observed that both receptors participate in the activation of DCs and secretion of important mediators involved in the development of the immune response. However, only TLR2 is required for neutrophil function. TLR9^−/−^ neutrophils presented a normal activation profile during infection, while TLR2^−/−^ neutrophils presented a defective effect on the expression of CD11b, an integrin expressed on the surface of neutrophils. This evidence demonstrates that TLRs play different roles depending on cell type expression during *L. infantum* infection, reinforcing the relevance of studies regarding TLR function in different cells during leishmaniasis. Interesting, we observed that the absence of TLR2 in macrophages do not interfere in the iNOS expression during *L. infantum* infection may be due the compensation of others TLR expression on such cells. In fact, Marcato et al. have demonstrated that NO production by macrophages can be independent on TLR2 (Marcato et al., 2008) and dependent on TLR4. Macrophages are equipped with a broader and greater levels of TLRs to perform their functions, such as production of mediators, phagocytosis, and presentation of antigens (Applequist et al., 2002).

The electron microscopy images demonstrate that after iv. inoculation of *L. infantum*, parasites migrate to target organs and are located inside the macrophages and neutrophils. After 1 h of infection, the intraneutrophil protozoans lost their ultrastructural integrity (Rousseau et al., 2001). These cells act through an array of microbicidal mechanisms. Among these mechanisms, NO production by iNOS expression is one of the most effective mechanisms for controlling *Leishmania* parasites (Liew et al., 1991; Wei et al., 1995). Our data demonstrated that during *L. infantum* infection, TLR2 signaling is important for inducing iNOS expression, particularly on neutrophils. Several signals are involved in iNOS expression, such as inflammatory cytokines (IFN-γ and TNF-α) and TLR ligands (Gao et al., 1999; Mühl et al., 2011). In the context of *Leishmania* infection, MyD88^−/−^, TLR9^−/−^, and TLR4^−/−^ mice presented deficient expression of iNOS in the lesion (Kropf et al., 2004; De Trez et al., 2009). However, it is not possible to affirm that the reduced iNOS expression observed in these deficient mice is due to a direct defect of TLR signaling on phagocytic cells and/or due to an impaired Th1 response. The defect in IFNγ and IL-17 production in *L. infantum* infected TLR2^−/−^ mice could compromise iNOS expression and NO production. In addition to that, our data shows that TLR2^−/−^ neutrophils presented a reduction in iNOS expression and uptake of *L. infantum* parasites even when primed with IFN-γ, demonstrating for the first time the relevance of TLR2 signaling on neutrophil function. Indeed, the absence of TLR2 increases IL-10 production by *in vitro*-infected neutrophils. It is well-known that iNOS expression is inhibited by IL-10 production in an autocrine or paracrine manner. IL-10 induces arginase 1 expression, which could inhibit iNOS activity through competition for their common substrate, arginine (Qualls et al., 2010).

Therefore, we conclude that TLR2 signaling plays an important role in immune protection against *L. infantum* infection. Mechanistically, DCs are activated during infection in a TLR2-dependent manner, promoting prototypal Th1 and Th17 subsets and CXCL1 production. Together, both Th subsets may promote neutrophil accumulation and migration to targeted organs. Furthermore, TLR2 has a direct effect on neutrophils, mediating their activation, NO and TNF production and *L. infantum* uptake.

## Author contributions

Conceived and designed the experiments: LS and VC. Performed the experiments: LS, Jd, Md, and PS. Analyzed the data: LS, RA, FC, JS, and VC. Contributed reagents/materials/analysis tools: RA, FC, JS, and VC. Wrote the paper: LS and VC.

### Conflict of interest statement

The authors declare that the research was conducted in the absence of any commercial or financial relationships that could be construed as a potential conflict of interest.

## References

[B1] Akira S. Uematsu S. Takeuchi O. (2006). Pathogen recognition and innate immunity. Cell 124, 783–802. 10.1016/j.cell.2006.02.015 16497588

[B2] Alexander J. Bryson K. (2005). T helper (h) 1/Th2 and *Leishmania*: paradox rather than paradigm. Immunol. Lett. 99, 17–23. 10.1016/j.imlet.2005.01.009 15894106

[B3] Applequist S. E. Wallin R. P. Ljunggren H. G. (2002). Variable expression of Toll-like receptor in murine innate and adaptive immune cell lines. Int. Immunol. 14, 1065–1074. 10.1093/intimm/dxf069 12202403

[B4] Ashour D. S. (2015). Toll-like receptor signaling in parasitic infections. Expert Rev. Clin. Immunol. 11, 771–780. 10.1586/1744666X.2015.1037286 25896399

[B5] Assis R. R. Ibraim I. C. Noronha F. S. Turco S. J. Soares R. P. (2012). Glycoinositolphospholipids from *Leishmania braziliensis* and *L. infantum:* modulation of innate immune system and variations in carbohydrate structure. PLoS Negl. Trop. Dis. 6:e1543. 10.1371/journal.pntd.0001543 22389743 PMC3289616

[B6] Bandyopadhyay S. Kar Mahapatra S. Chowdhury B. P. Jha M. K. Das S. Halder K.. (2015). Toll-like receptor 2 targeted rectification of impaired CD8^+^ T cell functions in experimental *Leishmania donovani* infection reinstates host protection. PLoS ONE 10:e0142800. 10.1371/journal.pone.0142800 26559815 PMC4641719

[B7] Becker I. Salaiza N. Aguirre M. Delgado J. Carrillo-Carrasco N. Kobeh L. G.. (2003). *Leishmania* lipophosphoglycan (LPG) activates NK cells through toll-like receptor-2. Mol. Biochem. Parasitol. 130, 65–74. 10.1016/S0166-6851(03)00160-9 12946842

[B8] Carlsen E. D. Liang Y. Shelite T. Walker D. Melby P. Soong L. (2015). Permissive and protective roles for neutrophils in leishmaniasis. Clin. Exp. Immunol. 182, 109–118. 10.1111/cei.12674 26126690 PMC4608500

[B9] Carregaro V. Valenzuela J. G. Cunha T. M. Verri W. A. Grespan R. Matsumura G.. (2008). Phlebotomine salivas inhibit immune inflammation-induced neutrophil migration via an autocrine DC-derived PGE2/IL-10 sequential pathway. J. Leukoc. Biol. 84, 104–114. 10.1189/jlb.1107797 18390928 PMC3178508

[B10] Chowdhury B. P. Bandyopadhyay S. Das S. Majumder S. Jha M. K. Majumdar S. B.. (2015a). The host-protective effect of Arabinosylated Lipoarabinomannan against *Leishmania donovani* infection Is associated with restoration of IFN-γ responsiveness. PLoS ONE 10:e0117247. 10.1371/journal.pone.0117247 25658110 PMC4319725

[B11] Chowdhury B. P. Das S. Majumder S. Halder K. Ghosh S. Biswas S.. (2015b). Immunomodulation of host-protective immune response by regulating Foxp3 expression and treg function in *Leishmania*-infected BALB/c mice: critical role of IRF1. Pathog. Dis. 73:ftv063. 10.1093/femspd/ftv063 26297915 PMC4626582

[B12] De Trez C. Magez S. Akira S. Ryffel B. Carlier Y. Muraille E. (2009). iNOS-producing inflammatory dendritic cells constitute the major infected cell type during the chronic *Leishmania major* infection phase of C57BL/6 resistant mice. PLoS Pathog. 5:e1000494. 10.1371/journal.ppat.1000494 19557162 PMC2695779

[B13] de Veer M. J. Curtis J. M. Baldwin T. M. DiDonato J. A. Sexton A. McConville M. J.. (2003). MyD88 is essential for clearance of *Leishmania major*: possible role for lipophosphoglycan and Toll-like receptor 2 signaling. Eur. J. Immunol. 33, 2822–2831. 10.1002/eji.200324128 14515266

[B14] Engwerda C. R. Kaye P. M. (2000). Organ-specific immune responses associated with infectious disease. Immunol. Today 21, 73–78. 10.1016/S0167-5699(99)01549-2 10652464

[B15] Franklin B. S. Ishizaka S. T. Lamphier M. Gusovsky F. Hansen H. Rose J.. (2011). Therapeutical targeting of nucleic acid-sensing Toll-like receptors prevents experimental cerebral malaria. Proc. Nat. Acad. Sci. U.S.A. 108, 3689–3694. 10.1073/pnas.1015406108 21303985 PMC3048158

[B16] Freitas-Silva R. Brelaz-de-Castro M. C. Rezende A. M. Pereira V. R. (2014). Targeting dendritic cells as a good alternative to combat *Leishmania* spp. Front. Immunol. 5:604. 10.3389/fimmu.2014.00604 25505469 PMC4245024

[B17] Gao J. J. Zuvanich E. G. Xue Q. Horn D. L. Silverstein R. Morrison D. C. (1999). Cutting edge: bacterial DNA and LPS act in synergy in inducing nitric oxide production in RAW 264.7 macrophages. J. Immunol. 163, 4095–4099. 10510342

[B18] Gatto M. de Abreu M. M. Tasca K. I. de Assis Golim M. da Silva L. D. M. Simão J. C.. (2015). The involvement of TLR2 and TLR4 in cytokine and nitric oxide production in Visceral Leishmaniasis patients before and after treatment with anti-leishmanial drugs. PLoS ONE 10:e0117977. 10.1371/journal.pone.0117977 25706930 PMC4338033

[B19] Ghosh K. Sharma G. Saha A. Kar S. Das P. K. Ukil A. (2013). Successful therapy of visceral Leishmaniasis with curdlan involves T-helper 17 cytokines. J. Infect. Dis. 207, 1016–1025. 10.1093/infdis/jis771 23255562

[B20] Hernández-Ruiz J. Salaiza-Suazo N. Carrada G. Escoto S. Ruiz-Remigio A. Rosenstein Y.. (2010). CD8 cells of patients with diffuse cutaneous leishmaniasis display functional exhaustion: the latter is reversed, *in vitro*, by TLR2 agonists. PLoS Neglected Trop. Dis. 4:871. 10.1371/journal.pntd.0000871 21072232 PMC2970528

[B21] Huang L. Hinchman M. Mendez S. (2015). Coinjection with TLR2 Agonist Pam3CSK4 reduces the pathology of Leishmanization in mice. PLoS Negl. Trop. Dis. 9:e0003546. 10.1371/journal.pntd.0003546 25738770 PMC4354918

[B22] Janssens S. Beyaert R. (2003). Role of Toll-like receptors in pathogen recognition. Clin. Microbiol. Rev. 16, 637–646. 10.1128/CMR.16.4.637-646.2003 14557290 PMC207104

[B23] Kavoosi G. Ardestani S. Kariminia A. (2009). The involvement of TLR2 in cytokine and reactive oxygen species (ROS) production by PBMCs in response to *Leishmania major* phosphoglycans (PGs). Parasitology 136, 1193–1199. 10.1017/S0031182009990473 19631014

[B24] Kavoosi G. Ardestani S. K. Kariminia A. Alimohammadian M. H. (2010). *Leishmania major* lipophosphoglycan: discrepancy in toll-like receptor signaling. Exp. Parasitol. 124, 214–218. 10.1016/j.exppara.2009.09.017 19769970

[B25] Komai-Koma M. Li D. Wang E. Vaughan D. Xu D. (2014). Anti-Toll-like receptor 2 and 4 antibodies suppress inflammatory response in mice. Immunology 143, 354–362. 10.1111/imm.12312 24801735 PMC4212949

[B26] Kropf P. Freudenberg M. A. Modolell M. Price H. P. Herath S. Antoniazi S.. (2004). Toll-like receptor 4 contributes to efficient control of infection with the protozoan parasite *Leishmania major*. Infect. Immun. 72, 1920–1928. 10.1128/IAI.72.4.1920-1928.2004 15039311 PMC375159

[B27] Liew F. Y. Li Y. Moss D. Parkinson C. Rogers M. V. Moncada S. (1991). Resistance to *Leishmania major* infection correlates with the induction of nitric oxide synthase in murine macrophages. Eur. J. Immunol. 21, 3009–3014. 10.1002/eji.1830211216 1721024

[B28] Liew F. Y. Wei X. Q. Proudfoot L. (1997). Cytokines and nitric oxide as effector molecules against parasitic infections. Philos. Trans. R. Soc. Lond. B Biol. Sci. 352, 1311–1315. 10.1098/rstb.1997.0115 9355122 PMC1692019

[B29] Liu D. Uzonna J. E. (2012). The early interaction of *Leishmania* with macrophages and dendritic cells and its influence on the host immune response. *Front. Cell*. *Infect*. Microbiol. 2:83 10.3389/fcimb.2012.00083 PMC3417671 22919674

[B30] Maisonneuve C. Bertholet S. Philpott D. J. De Gregorio E. (2014). Unleashing the potential of NOD-and Toll-like agonists as vaccine adjuvants. Proc. Natl. Acad. Sci. U.S.A. 111, 12294–12299. 10.1073/pnas.1400478111 25136133 PMC4151741

[B31] Marcato L. Ferlini A. Bonfim R. Ramos-Jorge M. Ropert C. Afonso L.. (2008). The role of Toll-like receptors 2 and 4 on reactive oxygen species and nitric oxide production by macrophage cells stimulated with root canal pathogens. Oral Microbiol. Immunol. 23, 353–359. 10.1111/j.1399-302X.2008.00432.x 18793356

[B32] Martins G. A. Petkova S. B. Machado F. S. Kitsis R. N. Weiss L. M. Wittner M.. (2001). Fas–FasL interaction modulates nitric oxide production in Trypanosoma cruzi-infected mice. Immunology 103, 122–129. 10.1046/j.1365-2567.2001.01216.x 11380700 PMC1783222

[B33] McConville M. Schnur L. Jaffe C. Schneider P. (1995). Structure of *Leishmania* lipophosphoglycan: inter-and intra-specific polymorphism in Old World species. Biochem. J. 310, 807–818. 10.1042/bj3100807 7575413 PMC1135969

[B34] McConville M. Turco S. Ferguson M. Sacks D. (1992). Developmental modification of lipophosphoglycan during the differentiation of *Leishmania major* promastigotes to an infectious stage. EMBO J. 11, 3593. 1396559 10.1002/j.1460-2075.1992.tb05443.x PMC556818

[B35] McFarlane E. Perez C. Charmoy M. Allenbach C. Carter K. C. Alexander J.. (2008). Neutrophils contribute to development of a protective immune response during onset of infection with *Leishmania donovani*. Infect. Immun. 76, 532–541. 10.1128/IAI.01388-07 18056477 PMC2223441

[B36] Mühl H. Bachmann M. Pfeilschifter J. (2011). Inducible NO synthase and antibacterial host defence in times of Th17/Th22/T22 immunity. Cell. Microbiol. 13, 340–348. 10.1111/j.1462-5822.2010.01559.x 21199257

[B37] Muraille E. De Trez C. Brait M. De Baetselier P. Leo O. Carlier Y. (2003). Genetically resistant mice lacking MyD88-adapter protein display a high susceptibility to *Leishmania major* infection associated with a polarized Th2 response. J. Immunol. 170, 4237–4241. 10.4049/jimmunol.170.8.4237 12682257

[B38] Nascimento M. S. L. Carregaro V. Lima-Júnior D. S. Costa D. L. Ryffel B. Duthie M.. (2014). IL-17A acts synergistically with IFN-γ to promote protection against *Leishmania infantum* infection. J. Infect. Dis. 211, 1015–1026. 10.1093/infdis/jiu531 25274569

[B39] Olekhnovitch R. Bousso P. (2015). Induction, propagation, and activity of host nitric oxide: lessons from *Leishmania* infection. Trends Parasitol. 31, 653–664. 10.1016/j.pt.2015.08.001 26440786

[B40] Qualls J. E. Neale G. Smith A. M. Koo M.-S. DeFreitas A. A. Zhang H.. (2010). Arginine usage in mycobacteria-infected macrophages depends on autocrine-paracrine cytokine signaling. Sci. Signal. 3, ra62. 10.1126/scisignal.2000955 20716764 PMC2928148

[B41] Raman V. S. Bhatia A. Picone A. Whittle J. Bailor H. R. O'Donnell J.. (2010). Applying TLR synergy in immunotherapy: implications in cutaneous leishmaniasis. J. Immunol. 185, 1701–1710. 10.4049/jimmunol.1000238 20601594 PMC3109724

[B42] Roses R. E. Xu S. Xu M. Koldovsky U. Koski G. Czerniecki B. J. (2008). Differential production of IL-23 and IL-12 by myeloid-derived dendritic cells in response to TLR agonists. J. Immunol. 181, 5120–5127. 10.4049/jimmunol.181.7.5120 18802116

[B43] Rousseau D. Demartino S. Ferrua B. Michiels J. F. Anjuère F. Fragaki K.. (2001). *In vivo* involvement of polymorphonuclear neutrophils in *Leishmania infantum* infection. BMC Microbiol. 1:17. 10.1186/1471-2180-1-17 11591218 PMC57739

[B44] Sabroe I. Prince L. R. Jones E. C. Horsburgh M. J. Foster S. J. Vogel S. N.. (2003). Selective roles for Toll-like receptor (TLR) 2 and TLR4 in the regulation of neutrophil activation and life span. J. Immunol. 170, 5268–5275. 10.4049/jimmunol.170.10.5268 12734376

[B45] Sacramento L. Trevelin S. C. Nascimento M. S. Lima-Junior D. S. Costa D. L. Almeida R. P.. (2015). Toll-like receptor 9 signaling in dendritic cells regulates neutrophil recruitment to inflammatory foci following *Leishmania infantum* infection. Infect. Immun. 83, 4604–4616. 10.1128/IAI.00975-15 26371124 PMC4645391

[B46] Smelt S. C. Cotterell S. E. J. Engwerda C. R. Kaye P. M. (2000). B cell-deficient mice are highly resistant to *Leishmania donovani* infection, but develop neutrophil-mediated tissue pathology. J. Immunol. 164, 3681–3688. 10.4049/jimmunol.164.7.3681 10725726

[B47] Stanley A. C. Engwerda C. R. (2006). Balancing immunity and pathology in visceral leishmaniasis. Immunol. Cell Biol. 85, 138–147. 10.1038/sj.icb7100011 17146466

[B48] Swihart K. Fruth U. Messmer N. Hug K. Behin R. Huang S.. (1995). Mice from a genetically resistant background lacking the interferon γ receptor are susceptible to infection with *Leishmania major* but mount a polarized T helper cell 1-type CD4^+^ T cell response. J. Exp. Med. 181, 961–971. 10.1084/jem.181.3.961 7869054 PMC2191906

[B49] Thalhofer C. J. Chen Y. Sudan B. Love-Homan L. Wilson M. E. (2011). Leukocytes infiltrate the skin and draining lymph nodes in response to the protozoan *Leishmania infantum* chagasi. Infect. Immun. 79, 108–117. 10.1128/IAI.00338-10 20937764 PMC3019875

[B50] Tuon F. F. Amato V. S. Bacha H. A. AlMusawi T. Duarte M. I. Neto V. A. (2008). Toll-like receptors and leishmaniasis. Infect. Immun. 76, 866–872. 10.1128/IAI.01090-07 18070909 PMC2258802

[B51] von Stebut E. Belkaid Y. Nguyen B. V. Cushing M. Sacks D. L. Udey M. C. (2000). *Leishmania major*-infected murine Langerhans cell-like dendritic cells from susceptible mice release IL-12 after infection and vaccinate against experimental cutaneous Leishmaniasis. Eur. J. Immunol. 30, 3498–3506. 10.1002/1521-4141(2000012)30:12<3498::AID-IMMU3498>3.0.CO;2-6 11093169

[B52] Wei X. Q. Charles I. G. Smith A. Ure J. Feng G. J. Huang F. P.. (1995). Altered immune responses in mice lacking inducible nitric oxide synthase. Nature 375, 408–411. 10.1038/375408a0 7539113

